# Cystoscopy of the Serous Cavities

**Published:** 1911-05

**Authors:** 


**Cystoscopy of the Serous Cavities.**

Dr. H. C. Jacobeus (*The Hospital,* Nov., 1910,) has conceived the idea of making a direct inspection of the peritoneal and pleural cavities by means of a cystoscope. He uses a trocar and canula, and a cystoscope of Nitze which is sufficiently small to pass within it. He first makes a small incision in the skin and passes in the trocar, empties the cavity of fluid, and insufflates air by means of a Politzer air-pump. He then introduces the cystoscope, which is fitted with a valve to prevent the air escaping. The maneuvre is easier in the case of the pleura than of the peritoneum, on account of the danger of injury to the viscera, but experiments on the cadaver have shown that the introduction of the trocar does not involve the risk of injury to the intestines, even when there is no ascites. The author has been able by this means to observe the presence of hepatic cancerous nodules, of perihepatitis, peritoneal congestion, cancerous nodules of the intestine, and to study intestinal peristalsis. In the case of the pleura he has not been able to detect definite lesions, but hopes to succeed in doing so with more dexterity. He is of opinion that "laparoscopy" and "thoracoscopy" should furnish useful data, comparable in some degree with those of exploratory laparatomy. Considering, however, the somewhat complicated and difficult technic and manipulation, and the very limited range of observation obtainable even in the most favorable circumstances, it may be doubted whether the method offers any but a very limited field of usefulness.

